# Current progress in cancer treatment by targeting FGFR signaling

**DOI:** 10.20892/j.issn.2095-3941.2023.0137

**Published:** 2023-07-24

**Authors:** Sicheng Du, Ying Zhang, Jianming Xu

**Affiliations:** 1 Department of Graduate Administration, Chinese People’s Liberation Army (PLA) General Hospital, Beijing 100853, China; 2 Department of Oncology, The Fifth Medical Center of the PLA General Hospital, Beijing 100071, China

Fibroblast growth factor receptors (FGFRs), a family of transmembrane receptors with intracellular tyrosine kinase domains, and fibroblast growth factors (FGFs) form the FGF/FGFR signaling pathways, which participate in cell development, differentiation, cell survival, migration, angiogenesis, and carcinogenesis. The FGF/FGFR family consists of 4 FGFRs and 22 ligands (FGFs). FGFR binding to cognate ligands induces receptor dimerization and intracellular phosphorylation of receptor kinase domains. As a result, four downstream intracellular pathways are triggered: RAS-RAF-MEK-MAPK; PI3K-AKT-mTOR; JAK-STAT; and PLCγ (**Figure 1**). Overactivation of the RAS-RAF-MEK-MAPK pathway stimulates cell proliferation and differentiation, while PI3K-AKT-mTOR pathway overactivation inhibits apoptosis. The JAK-STAT pathway promotes tumor invasion and metastasis, and enhances tumor immune evasion. The PLCγ signaling pathway has an important role in regulating tumor cell metastasis. Alterations in FGFR genes, including gene amplification, activating mutations, rearrangements, and fusions, can result in excessive activation of the FGFR signaling pathway and further induce normal cell carcinogenesis. In this review we summarize the types of FGFR aberrations and advances in drugs targeting the FGF/FGFR pathway. We also comment on potentially effective strategies and current obstacles in anti-FGFR therapy.

**Figure 1:**
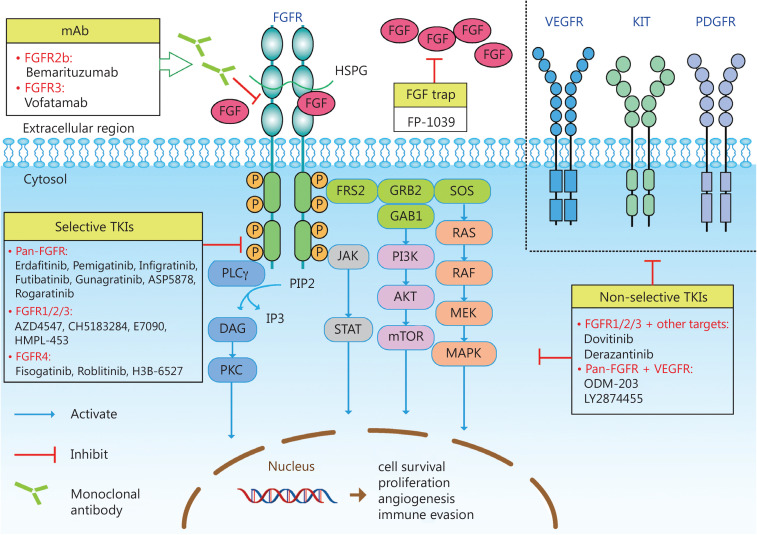
FGFR signaling and inhibitors. The family of FGF receptors (FGFR1-4) are receptor tyrosine kinases expressed on cell membranes with significant sequence homology. Each FGFR typically consists of three extracellular immunoglobulin-like domains, a hydrophobic transmembrane domain, and two intracellular tyrosine kinase (TK) domains. FGF and FGFR binding stimulates receptor dimerization. This interaction can be stabilized by heparan sulfate proteoglycans (HSPGs). FGF-FGFR binding further phosphorylates intracellular FGFR substrate 2 (FRS2), phospholipase C gamma (PLCγ), and JAK, thereby activating four major signaling pathways. (1) The activation of FRS2 recruits the adaptor proteins [GRB2 and son of sevenless (SOS)], which results in subsequent activation of MAPK. (2) GRB2 recruits GAB1, which leads to activation of the PI3K-AKT-mTOR pathway. (3) Phosphorylation of PLCγ hydrolyzes phosphatidylinositol 4,5-bisphosphate (PIP2) to phosphatidylinositol 3,4,5-tri-phosphate (IP3) and diacylglycerol (DAG), thus activating protein kinase C (PKC). (4) JAK-STAT signaling can also be activated. FGF/FGFR pathway inhibitors are mainly divided into mAb/FGF trap, which prevent FGF and FGFR binding in the extracellular domain, and small molecule TK inhibitors (TKIs) that target the ATP-binding cleft of TK domains inside the cell. Selective TKIs specifically target the FGFR kinase domains, while non-selective TKIs target several phylogenetically-related growth factor receptors, such as VEGFR, KIT, and PDGFR. JAK, Janus kinase; STAT, signal transducer and activator of transcription; GRB2, growth factor receptor-bound protein 2; GAB1, GRB2-associated binding protein 1; VEFGR, vascular endothelial growth factor receptor; PDFGR, platelet-derived growth factor receptor.

## FGFR aberrations and distribution

FGFR aberrations are widely distributed in all malignant tumors, including urothelial carcinoma (32%), breast cancer (18%), endometrial cancer (13%), lung squamous cell carcinoma (13%), and ovarian cancer (9%), with an overall frequency of 7.1%, The major type of aberration is amplification (66%), followed by mutations (26%), rearrangements (8%), and fusions (< 1%)^1^. The proportions of FGFR1-4 aberrations are 3.5%, 1.5%, 2.0%, and 0.5%^1^, respectively (**Table 1**).

**Table 1:** FGFR alteration types and frequency

Target (alteration rate %)	Aberration (frequency %)	Cancer (frequency %**)
FGFR1 (3.5%)	Amplification (89%) Mutation (8%) Other* (2%)	Breast (13.8%), urothelial (8.7%), ovarian/fallopian (4.7%), neuroendocrine (3.7%), glioma (3.5%), non-small cell lung (2.7%), sarcoma (2.5%), colorectal (2.4%)
FGFR2 (1.5%)	Amplification (49%) Mutation (18%) Fusion (15%) Other* (18%)	Endometrial (7.5%), cholangiocarcinoma (6.1%), gastric/GE junction (3.7%), breast (2.3%)
FGFR3 (2.0%)	Amplification (30%) Mutation (44%) Fusion (15%) Other* (11%)	Urothelial (22.2%), glioma (4.2%), endometrial (2.5%), pancreatic exocrine (2.3%), renal cell (2.3%)
FGFR4 (0.5%)	Amplification (78%) Other* (22%)	Renal cell (1.1%), non-small cell lung (1.0%)

*Other rare aberrations. **Cancers with a frequency < 2% are not listed in the table. GE, Gastroesophageal.

### FGF/FGFR amplification and overexpression

Amplification is the most common FGFR1 alteration and is prevalent in patients with breast and non-small cell lung cancers. FGFR1 amplification has been shown to be associated with endocrine resistance and suppression of progesterone receptor expression in patients with hormone receptor-positive breast cancer (HR+BC), while FGFR1 blockade appears to revert endocrine resistance in cell lines with amplification and concomitant overexpression of FGFR1^2^. Of note, FGFR1 amplification and FGFR1 mRNA overexpression do not always correlate. Poor concordance between FGFR1 amplification, as determined by fluorescence *in situ* hybridization (FISH), and FGFR1 mRNA overexpression, as determined by RNAscope, has recently been reported in > 20% of HR+BC patients^3^. FGFR2 amplification is less frequent than FGFR1 amplification across cancer types and is most often reported in patients with gastric-oesophageal junction adenocarcinoma and breast cancer. Unlike FGFR1, FGFR2 inhibition has relevant activity in high FGFR2-amplified cell lines, suggesting addiction to the FGFR pathway. Oncogene addiction for FGFR2 amplification is more pronounced than FGFR1 amplification, which has been attributed to the different amplicon structure. FGFR2 amplicons are generally narrow and centred on FGFR2, with few other genes co-amplified, whereas the amplicon structure of FGFR1 is often broad, co-amplified by multiple genes, and has a stronger oncogenic effect^4^, thus resulting in FGFR1 inhibitor inefficacy.

Amplification of FGFR3 and FGFR4 has rarely been reported. While amplification of FGF3, FGF4, FGF19, and CCND1, which are all located on chromosome 11q13, has been detected in several cancer types but rarely results in FGFs overexpression. FGF19 gene amplification has been shown to increase the risk of cirrhosis. And FGF19 overexpression, which exists in 15% of hepatocellular carcinoma (HCC) patients with amplification of the 11q13 locus, has been proved to be highly associated with carcinogenesis^5^. FGF19, the main functions of which are bile acid synthesis, gallbladder filling, glycogen synthesis, gluconeogenesis, and protein synthesis, binds FGFR4 with the highest affinity of three endogenous fibroblast growth factors (FGF19, FGF21, and FGF23). Overexpression of paracrine/autocrine FGF19 causes FGF19/FGFR4/KLB activation, leading to the formation of FGF receptor substrate 2 (FRS2) and growth factor receptor-binding protein 2 (GRB2) complexes, which ultimately activate the Ras-Raf-MAPK and PI3K-Akt pathways^6^. RNA interference-mediated knockdown and neutralizing antibodies against FGF19 have a profound anti-proliferative effect on HCC in *in vitro* and *in vivo* models. These results suggest that FGF19/FGFR4 inhibition leads to anti-tumor activities and may be a potential target in HCC. Overexpression of several other FGFs has also been shown to induce carcinogenesis and promote tumor progression in murine studies. Specifically, conditional FGF10 expression in lung epithelium induces pulmonary tumors^7^ and FGF8 overexpression in prostate epithelium is associated with a high risk of prostate cancer^8^. Moreover, FGF1 overexpression, following FGF1 gene amplification, is associated with poor survival in patients with ovarian cancer^9^. Based on the above discussion, drugs that bind FGFs extracellularly, like FGF ligand traps, may be a potential therapeutic strategy for FGF-overexpressed malignancies.

### FGFR mutations

Mutations in FGFR2 and FGFR3 are common, while FGFR1 and FGFR4 mutations are rarely observed. In contrast to epidermal growth factor receptor (EGFR) mutations, somatic activating mutations of FGFR are mostly outside the kinase domain. Furthermore, mutations in the kinase domain of FGFR1 (most frequently N546K), FGFR2 (most frequently N549H/K) and FGFR4 (most frequently K535 and E550), although rarely reported, can also directly increase kinase activity and induce cell transformation. FGFR2 mutations occur most frequently in endometrial cancer (EC) (10%–12%); however, not all FGFR2 mutations are effective targets. Dovitinib, a non-selective inhibitor, did not reach the prespecified study criteria in the second-line treatment of EC. The objective response rate (ORR) was reported to be 4.5% in the FGFR2-mutated group and 16.1% in the group without FGFR2 mutations^10^. In patients with intrahepatic cholangiocarcinoma (iCCA) enrolled in the FIGHT-202 trial^11^, pemigatinib did not result in a favourable response in the group with FGFR2 mutations. In approximately 3% of iCCA patients, FGFR2 insertions/deletions generate in-frame deletions in the extracellular domain (IED), which can impact ligand recognition and/or receptor dimerization and ultimately lead to FGFR2 oncogenic activation linked to iCCA pathogenesis. An *in vitro* experiment demonstrated that exon 18 truncation of FGFR2 may be a potent driver mutation that increases the response rate to FGFR inhibitors in cancer cell lines and mouse models^12^. In contrast to IEDs, most sporadic FGFR2 point mutations may not generate levels of FGFR2 activity sufficient to drive pathogenesis and establish oncogenic dependence, which therefore results in clinical response inefficacy. FGFR3 mutations are present in approximately 75% of non-muscle invasive urothelial carcinomas (UCs) and 15% of muscle invasive UCs. FGFR3 mutations were significantly associated with lower pT stage, tumor grade, absence of carcinoma *in situ*, pN0, low level of p53, and longer disease-specific survival (DSS) based on an analysis of 1000 bladder cancer resection specimens^13^. FGFR3 overexpression was only associated with lower pT stage and tumor grade. Patients with bladder cancer and FGFR3 mutations have a favourable prognosis and are more likely to benefit from anti-FGFR3 therapy compared to patients with FGFR3-overexpressing tumors^13^.

### FGFR rearrangements and fusions

FGFR gene rearrangements are DNA structural alterations that produce chimeric fusion proteins comprised of the fusion partner gene portion bound to the FGFR kinase domain if transcriptionally active with a preserved reading frame. Oncogenic FGFR fusions have been identified in several cancers, in which FGFR2 and FGFR3 fusions are mostly observed. FGFR2 fusions occur almost exclusively in iCCA, with a frequency of 10%–15%, and are rare in extrahepatic cholangiocarcinoma (CCA) and other epithelial cancers. The most common partner of FGFR2 is BICC1; other partners include greater than 40 kinds of fusion partners, such as CIT, CCDC6, CCAR2, OFD, AHCYL1, and PPHLN1^4^. Most of the FGFR2 partners contain dimeric domains and can induce ligand-independent receptor dimerization and oncogenic effects. FGFR2 fusions in iCCA have been associated with a better prognosis and younger age at diagnosis in some studies^14^ and have been shown to be frequently co-altered with mutations in the chromatin-remodeling gene, BAP1^14^, a tumor suppressor in iCCA.

FGFR3 fusions are relatively common in patients with glioblastomas and bladder cancer, but rarely reported in patients with lung cancer. The fusion partner of FGFR3 is commonly known as TACC3^4^; the C-terminal exon of FGFR3 is replaced by TACC3, thus making FGFR3 carcinogenic. The fusion protein can promote cell proliferation by increasing MAPK-ERK and JAK-STAT pathway activation, while FGFR inhibitors exert anti-tumor effects by downregulating signal transduction.

## Clinical application of FGFR inhibitors

Currently, drugs targeting the FGF/FGFR signaling pathway in clinical practice mainly include non-selective tyrosine kinase inhibitors (TKIs), selective TKIs (pan-FGFR, FGFR1/2/3, and FGFR4 inhibitors), monoclonal antibodies, and FGF ligand traps (**Table 2**). The efficacy of the same drug varies significantly among different tumor types, which may be related to tumor heterogeneity; however, the specific underlying mechanism has not been established.

**Table 2:** Summary of FGFR inhibitors currently under investigation in clinical trials

Inhibitor (drug ID)	Targets	IC50 (nM)	Clinical trials	Cancer types	Results
Non-selective inhibitors					
Dovitinib (TKI258)	FGFR1/2/3; KIT; VEGFR	FLT3: 1, KIT: 2, FGFR1: 8, VEGFR3: 8, FGFR3: 9, VEGFR1: 10	Phase II NCT01861197	FGFR1-amplified squamous NSCLC	ORR: 11.5%; DCR: 50%; mPFS: 2.9 m; mOS: 5.0 m
			Phase II NCT01379534	FGFR2-mutated or WT EC	ORR: 4.5% *vs*. 16.5%; DCR: 63.6% *vs*. 51.6%; PFS: 4.1 m *vs*. 2.7 m; 18-week PFS rate: 31.8% *vs*. 29%; mOS: 20.2 m *vs*. 9.3 m (FGFR2 mutation *vs*. WT)
			Phase III NCT01223027	Metastatic renal cell cancer	mPFS: 3.7 m *vs*. 3.6 m (Dovitinib *vs*. Sorafenib)
Derazantinib (ARQ-087)	FGFR1/2/3; KIT; VEGFR; PDGFRβ	FGFR2: 1.8, FGFR1: 4.5, FGFR3: 4.5, FGFR4: 34, RET: 3, DDR2: 3.6, VEGFR1: 11, KIT: 8.2	Phase I/II NCT01752920	FGFR aberrant advanced solid tumors	RP2D: 300 mg QD; ORR: 20.7%; DCR: 82.8%; mPFS: 5.7 m (all pts); mPFS: 7.95 m (FGFR2 fusion-positive pts)
ODM-203	Pan-FGFR; VEGFR1/2/3	FGFR1: 11, FGFR2: 16, FGFR3: 6, FGFR4: 35, VEGFR1: 26	Phase I/IIa NCT02264418	Advanced or metastatic solid tumors	ORR: 9.2%; mPFS: 16.1 and 12.4 weeks for aberrant or non-aberrant FGFR
LY2874455	Pan-FGFR; VEGFR2	FGFR1: 2.8, FGFR2: 2.6, FGFR3: 6.4, FGFR4: 6, VEGFR2: 7	Phase I NCT01212107	Advanced or metastatic solid tumors	RP2D: 16 mg BID
Selective inhibitors					
Erdafitinib (JNJ-42756493)	Pan-FGFR	FGFR1: 1.2, FGFR2: 2.5, FGFR3: 3.0, FGFR4: 5.7	*FDA approved* Phase II NCT02365597	FGFR2/FGFR3 aberrant UC	ORR: 40%; mPFS: 5.5 m; mOS: 13.8 m
			Phase I/IIa NCT02421185	FGF19-amplified HCC	ORR: 4.8%; DCR: 35.7% *vs*. 9.1%; m PFS: 1.58 m *vs*. 1.31 m (FGF19 amplifcation *vs*. no-FGF19 amplifcation)
Pemigatinib (INCB054828)	Pan-FGFR	FGFR1: 0.4, FGFR2: 0.5, FGFR3: 1.0, FGFR4: 30	*FDA approved* Phase II NCT02924376	FGFR2 fusions or rearrangement in CCA	ORR: 35.5%; m PFS: 6.9 m; mOS: 21.1 m; mDOR: 7.5 m; DCR: 82.0%
Infigratinib (BGJ398)	Pan-FGFR	FGFR1: 0.9, FGFR2: 1.4, FGFR3: 1.0, FGFR4: 60	*FDA approved* Phase II NCT02150967	FGFRs aberrant CCA	ORR: 14.8%; DCR: 75.4%; m PFS: 5.8 m
			Phase II NCT02160041	FGFRs aberrant malignancies	CBR: 15%; ORR: 7.5%; mPFS: 1.8 m; OS: 6.2 m
Futibatinib (TAS-120)	Pan-FGFR	FGFR1: 3.9, FGFR2: 1.3, FGFR3: 1.6, FGFR4: 8.3	*FDA approved* Phase II NCT02052778	FGFR2 fusions/rearrangement in iCCA	ORR: 42%; mPFS: 9.0 m; OS: 27.1 m
Gunagratinib (ICP-192)	Pan-FGFR	FGFR1: 1.4, FGFR2: 1.5, FGFR3: 2.6, FGFR4: 3.5	Phase I/IIa NCT03758664	FGFR2 translocation/fusion in CCA	CR: 8.4%, PR: 25.0%, SD: 58.3% (12 pts)
ASP5878	Pan-FGFR	FGFR1: 0.5, FGFR2: 0.6, FGFR3: 0.7, FGFR4: 3.5	Phase I NCT02038673	FGFRs aberrant UC,HCC or squamous NSCLC	NA
Rogaratinib (BAY-1163877)	Pan-FGFR	FGFR1: 1.8, FGFR2: < 1, FGFR3: 9.2, FGFR4: 1.2	Phase II/III NCT03410693	FGFR-positive UC processed with prior platinum-containing chemotherapy	ORR: 20.7% *vs*. 19.3%; mPFS: 8.3 m *vs*. 9.8 m (rogaratinib *vs*. chemotherapy)
AZD4547	FGFR1/2/3	FGFR1: 0.2, FGFR2: 2.5, FGFR3: 1.8	Phase II NCT02465060	FGFR1/2/3 aberrant tumuors	ORR: 8%, mPFS: 3.4 m, 6-month PFS rate: 15%; (For FGFR fusions patients, ORR: 22%, 6-month PFS: 56%)
			Phase II NCT01457846	FGFR2-amplified or polysomic GC	mPFS: 1.8 m *vs*. 3.5 m (AZD4547 *vs*. Paclitaxel)
CH5183284 (Debio-1347)	FGFR1/2/3	FGFR1: 9.3, FGFR2: 7.6, FGFR3: 22, FGFR4: 290	Phase I NCT01948297	FGFR1-3 altered solid malignancies	PR: 10%, SD: 27.5%, PD: 60% (58 pts)
			Phase II NCT03834220	FGFR 1/2/3 gene fusion or rearrangement in solid tumors	NA
E7090	FGFR1/2/3	FGFR2: 1.2	Phase I NCT02275910	Advanced solid tumors regardless of FGFR alteration	PR: 4%, SD: 29%, PD: 58% (24 pts)
			Phase II NCT04238715	FGFR2 gene fusions in CCA	NA
HMPL-453	FGFR1/2/3	FGFR1: 6, FGFR2: 4, FGFR3: 6	Phase II NCT04353375	FGFR2 fusions in advanced bile duct cancer	NA
Fisogatinib (BLU-554)	FGFR4	FGFR4: 5	Phase I NCT02508467	FGF19 positive advanced HCC	ORR: 17% *vs*. 0%; mPFS: 3.3 m *vs*. 2.3 m (FGF19-positive *vs*. FGF19-negative)
Roblitinib (FGF401)	FGFR4	FGFR4: 1.9	Phase I/II NCT02325739	FGF19 positive advanced HCC	RP2D: 120 mg QD; ORR: 8%, SD: 53%, mPFS: 4.1 m
H3B-6527	FGFR4	FGFR4: < 1.2	Phase I NCT02834780	Advanced HCC or iCCA	NA
Monoclonal antibodies and ligand traps					
Vofatamab	FGFR3	NA	Phase Ib/II NCT02401542	Vofatamab ± Docetaxel in UC	ORR: 11% (7/61)
			Phase Ib/II NCT03123055	Vofatamab + Pembrolizumab in UC	ORR: 11% (7/61)
Bemarituzumab (FPA114)	FGFR2b	NA	Phase II NCT0369452	FGFR2b overexpressed and/or FGFR amplified G/GEJC	mPFS: 9.5 m *vs*. 7.4 m; mOS: not reached *vs*. 12.9 m (mFOLFOX6 + bemarituzumab *vs*. mFOLFOX6 + placebo)
FP-1039	FGF2 trap	FGFR2: 0.023 μg/mL	Phase I NCT01868022	FGFR genetically aberrant solid malignancies	DLT: 0.75 mg/kg (urticaria), 1 mg/kg (intestinal perforation and neutropenia), and 16 mg/kg (muscular weakness)

NA, not available; NSCLC, non-small cell lung cancer; WT, wild type; EC, endometrial cancer; HCC, hepatocellular carcinoma; iCCA, intrahepatic cholangiocarcinoma; UC, urothelial carcinoma; GC, gastric cancer; G/GEJC, gastric/gastroesophageal junction cancer; ORR, objective response rate; DCR, disease control rate; mPFS, median progression-free survival; mOS, median overall survival; RP2D, recommended phase II dose; QD, once daily; BID, twice daily.

### Non-selective TKIs

Non-selective TKIs exert anti-tumor effects by inhibiting multiple FGFR kinase domains, vascular endothelial growth factor receptor (VEGFR), and platelet-derived growth factor receptor (PDGFR); however, the FGFR IC_50_ in non-selective TKIs has no apparent advantage over other targets and is generally higher than selective FGFR inhibitors. The inhibitory effect of non-selective TKIs on the FGF/FGFR pathway has been insignificant in clinical studies, but the dose-limiting toxicity of other targets, such as hypertension, has been observed.

### Selective TKIs

Selective TKIs only inhibit the FGFR pathway, thus avoiding the toxic effects of other targets. The FGFR1-3 kinase domains are highly similar, whereas FGFR4 has a unique structure. Most selective inhibitors inhibit FGFR1-3 to varying degrees, while a few TKIs only inhibit FGFR4.

#### Pan-FGFR inhibitors

Erdafitinib (JNJ-42756493) is an FGFR1–4 inhibitor with potent tyrosine kinase inhibitory activity against all four FGFR family members. The U.S. Food & Drug Administration (FDA) approved erdafitinib for patients with locally advanced or metastatic UC with susceptible FGFR3 or FGFR2 genetic alterations that progressed during or following platinum-containing chemotherapy based on a phase II study (NCT02365597). Among 99 enrolled patients, 3 (3%) achieved a complete response (CR), 37 (37%) had a partial response (PR), and 39 (39%) had stable disease (SD). The median progression-free survival (PFS) and overall survival (OS) were 5.5 and 13.8 months, respectively^15^.

Infigratinib (BGJ398) is an oral ATP-competitive FGFR1–3 selective TKI with weak activity against FGFR4. A phase II study (NCT02150967)^16^ enrolled 108 previously-treated patients with FGFR1-3 altered CCAs; the most frequent alteration was an FGFR2 fusion (88/108), followed by other rearrangements in FGFR2 (20/108). Overall, 1 (1%), 24 (22%), and 66 (61%) patients were shown to have a CR, PR, and SD, respectively, while 11 (10%) patients had progressive disease (PD) as the best response; the mean (m)PFS and mOS was 7.3 and 12.2 months, respectively^16^. Based on these data, infigiratinib was granted approval for CCA by the FDA.

Pemigatinib (INCB054828) is an inhibitor of FGFR1-3 with weak activity against FGFR4. In the phase II study [FIGHT-202 (NCT02924376)]^11^, 38 [35.5% (3 with a CR and 35 with a PR)] of 107 patients with an FGFR2 fusion or rearrangement of a CCA achieved objective remission with oral pemigatinib. The median duration of response was 7.5 months, with 68% of patients in remission ≥ 6 months and 37% of patients in remission ≥ 12 months^11^. Based on this finding, pemigatinib was approved by the FDA for patients with an FGFR2 fusion or rearrangement with refractory, advanced CCA.

Futibatinib is an irreversible inhibitor of FGFR1–4. The FOENIX-CCA2 study^17^ reported encouraging outcomes in patients with FGFR2 fusion/rearrangement-positive iCCA. Among 103 enrolled patients, 43 (42%) had a response. The median duration of response (mDOR) was 9.0 months and the mOS was 21.7 months. Therefore, futibatinib gained FDA approval for iCCAs harbouring FGFR2 gene fusions or other rearrangements.

Gunagratinib (ICP-192) is a pan-FGFR inhibitor with FGFR1-4 IC_50_ values of 1.4, 1.5, 2.4 and 3.5 nM, respectively. In February 2021 the phase I/IIa trial (NCT03758664)^18^ recruited 12 previously-treated CCA patients with FGF/FGFR2 gene aberrations, of whom 1 (8.4%) had a CR, 3 (25.0%) had a PR, and 7 (58.3%) had SD after receiving gunagratinib treatment. Based on this finding, gunagratinib was granted an orphan drug designation in 2021 by the FDA for CCA. The latest updated data showed that the ORR was 52.9% (9/17) and the mPFS was 6.93 months^19^.

Other pan-FGFR inhibitors have shown clinical activity to some extent but warrant further research; the available data are listed in **Table 2**.

#### FGFR1/2/3 inhibitors

AZD4547 is an oral FGFR1-3 inhibitor with IC_50_ values of 0.2, 1.8 and 2.5 nM. A phase II study (NCT02465060) evaluated AZD4547 in 48 patients with FGFR1/2/3-altered tumors but failed to meet the primary endpoint with an observed ORR of 8%^20^. Moreover, AZD4547 did not demonstrate a significantly improved PFS compared to paclitaxel (1.8 months for AZD4547 *vs.* 3.5 months for paclitaxel) in patients with FGFR2-amplified or polysomic gastric cancer (GC) in the second-line setting in another phase II trial (NCT01457846)^21^.

Debio 1347 is an FCFR1-3 ATP competitive inhibitor with IC_50_ values of 9.3, 7.6 and 22 nM, respectively. A phase I trial (NCT01948297) enrolled 58 patients with FGFR1-3-altered solid malignancies in the dose escalation phase, of whom 6 (10.3%) had a PR, 16 (27.6%) had SD, and 35 (60.3%) had PD^22,23^. Response to therapy was consistent with FGFR alterations in this study. A phase II trial (NCT03834220) with Debio 1347 is underway.

E7090 is a reversible FGFR1-3 inhibitor. A dose escalation phase I trial (NCT02275910)^24^ enrolled 24 previously-treated patients with advanced solid tumors regardless of FGFR alteration. Of the 24 patients, 1 (4%) with FGFR2-amplified GC had a PR, 7 (29%) had SD, and 14 (58%) had PD as the best response. The expansion phase^25^ involved 16 patients with FGFR-altered tumors who received a daily dose of 140 mg. Among 6 patients with FGFR-altered CCA, 5 (83%) had a PR and 1 (17%) had SD as the best response with an 8.3-month reported mPFS, whereas 1 of 10 patients with GC had a PR.

#### FGFR4 inhibitors

Fisogatinib (BLU-554), an FGFR4-specific inhibitor, showed efficacy in FGF19-positive advanced HCC in a phase I trial (NCT02508467). The ORR was 17%, and the median PFS was 3.3 months in FGF19-positive patients versus 0% and 2.3 months in FGF19-negative patients^26^. Fisogatinib was granted an orphan drug designation in 2015 by the FDA for HCC.

Roblitinib (FGF401) is an FGFR4 inhibitor with an IC_50_ of 1.9 nM. A phase I/II trial (NCT02325739)^27^ showed a favourable activity profile for roblitinib at the fasting recommended phase 2 dose (RP2D) of 120 mg daily. This trial enrolled patients with HCC and other solid tumors with positive FGFR4 and KLB expression. Among 53 patients with HCC, 8% had an OR, 53% had SD, and the reported median time-to-progression was 4.1 months^27^.

Although FGFR TKIs have showed encouraging outcomes in some clinical trials, drug resistance, as occurs in other small molecule inhibitors, is inevitable. Acquired or intrinsic resistance to FGFR TKIs has been shown to be related to the gatekeeper mutations in FGFRs and activation of alternative receptor tyrosine kinases. Therefore, dual or multiple inhibition of FGFR and other receptors appears to be a potential strategy to overcome this problem. A recent study showed that the combination of lenvatinib and an EGFR TKI enhanced inhibition of proliferation in patients with liver cancer^28^. Moreover, combining endocrine agents and FGFR inhibitors has demonstrated promising outcomes in clinical studies of breast cancer (NCT03238196, NCT03344536, and NCT01528345). Several phase II studies are ongoing.

### Monoclonal antibodies

Monoclonal antibodies specifically target a particular FGF ligand or FGFR isoform and have shown anti-tumor activity in cancer cell lines and murine models. The efficacy of monoclonal antibody monotherapy is limited in *in vivo* experiments. Current treatment strategies are mostly inclined to combination therapy.

Vofatamab, a humanized FGFR3 monoclonal antibody, binds to the extracellular portion of FGFR3, thus inhibiting ligand interaction and dimerization. Combination therapy with docetaxel or pembrolizumab for UC showed good tolerance in phase Ib/II trials (NCT02401542)^29^ and (NCT03123055)^30^, and the ORRs were 11% (7/61) and 30% (6/35), respectively.

Bemarituzumab (FPA114) is a humanized IgG1 FGFR2b monoclonal antibody. In the FIGHT trial (NCT0369452)^31^, a total of 155 patients with gastric or gastro-oesophageal junction adenocarcinomas harbouring FGFR2b overexpression and/or FGFR amplifications were randomly assigned at a 1:1 ratio to mFOLFOX6 plus bemarituzumab or placebo. The mPFS in the bemarituzumab plus mFOLFOX6 group was 9.5 months versus 7.4 months in the mFOLFOX6 plus placebo group. The mOS was not reached in the mFOLFOX6 plus placebo group versus 12.9 months in the bemarituzumab plus mFOLFOX6 group. Patients with FGFR2b overexpression had substantially improved mPFS, including 10.2 and 14.1 months for those with FGFR2b overexpression on > 5% and > 10% of tumor cells, respectively, whereas FGFR2b expression did not affect PFS in the placebo group.

### FGF ligand traps

FGF traps are a group of structurally inhomogeneous molecules with the ability to act as FGFR bait by binding FGF in the extracellular environment, thereby preventing growth factors from interacting with target cells. FP-1039 is an FGF ligand trap containing the extracellular domain of FGFR1-IIIc splicing isoforms. Patients with metastatic or locally advanced solid tumors received FP-1039 treatment in a recent phase I study^32^, and the best response recorded was SD (41.7%) among 39 unselected patients. No relationship between anti-tumor effects and FGF pathway aberrations was observed.

## Conclusions

Targeted FGF/FGFR therapy has clearly progressed, but the response rate is lower than other driver-positive tumors. Drugs with the same target exhibit different clinical activities across tumor types. Moreover, the specific mechanism needs further exploration in the future. Qualitative alterations in FGFR1-3, such as mutations and rearrangements, are sensitive to FGFR inhibitors in various cancer types, whereas quantitative alterations, such as amplification, seem to be less effective targets. This finding may be related to the heterogeneity of tumors, redundancy of oncogenes in amplicons, and the low correlation between amplification and overexpression. Among current FGFR inhibitors under investigation, selective TKIs demonstrated better efficacy, and several were approved for clinical use. Compared with small molecule TKIs, monoclonal antibodies tend to be combined with chemotherapy or immunotherapy to enhance anti-tumor effects. Like other TKIs, the efficacy of FGFR inhibitors is limited by drug resistance. Gatekeeper mutations and alternative pathway signal activation are the key factors leading to drug resistance. Combination therapy can block multiple activated pathways at the same time and is expected to reverse drug resistance. The combination therapy strategy should be adjusted according to different tumor types and FGFR aberrations to mostly enhance efficacy and avoid toxicity, thus making treatment targeting FGFR signaling individualized and precise.
